# Supplementary material for: “Being a man is like being put in a box”: A qualitative study of adolescent boys’ and young men’s understanding and experiences of mental health in an urban community in South Africa
Source: PLOS Ment Health. 2026 Feb 6;3(2):e0000365. doi: 10.1371/journal.pmen.0000365 (PMC12880673; doi:10.1371/journal.pmen.0000365)
Supplement: S1 File — (DOCX) [file pmen.0000365.s001.docx]

# COREQ (Consolidated Criteria for Reporting Qualitative Research) Checklist

| **Item** | **Response** |
| --- | --- |
| **Research team and reflexivity** | |
| 1. Interviewer/facilitator: Which author/s conducted the interview or focus group? | Two interviewers: (1) former GRS youth mentor (Alexandra resident), (2) senior GRS staff/mental health advisor. |
| 2. Credentials: What were the researcher's credentials? (e.g., PhD, MD) | Public health and education backgrounds; training in qualitative methods. |
| 3. Occupation: What was their occupation at the time of the study? | GRS staff (mental health advisor); research assistant was a community-based educator. |
| 4. Gender: Was the researcher male or female? | One male researcher (American, 40s), one Black South African male researcher (local). |
| 5. Experience and training: What experience or training did the researcher have? | Both had training in qualitative research methods; supervised by Stellenbosch lecturer. |
| 6. Relationship established: Was a relationship established prior to study commencement? | Yes. Local interviewer was a former youth mentor, known in the community. |
| 7. Participant knowledge of the interviewer: What did the participants know about the researcher? | Participants knew interviewer backgrounds (staff/mentor). Adolescent boys recruited through GRS program. |
| 8. Interviewer characteristics: What characteristics were reported about the interviewer/facilitator? | Characteristics: Local interviewer shared community/age background; senior researcher outsider with positionality noted. |
| **Study Design** | |
| 9. Methodological orientation and theory: What methodological orientation was stated to underpin the study? | Reflexive thematic analysis (Braun & Clarke, 2006, 2019). |
| 10. Sampling: How were participants selected? | Purposive sampling. |
| 11. Method of approach: How were participants approached? | In-person, at GRS offices in Alexandra. |
| 12. Sample size: How many participants were in the study? | 24 (12 adolescent boys, 12 mentors/staff). |
| 13. Non-participation: How many people refused or dropped out? | None reported; all invited mentors/staff participated. |
| 14. Setting of data collection: Where was the data collected? | GRS offices, private rooms. |
| 15. Presence of non-participants: Was anyone else present besides the participants and researchers? | No non-participants present. |
| 16. Description of sample: What are the important characteristics of the sample? | Adolescent boys aged 15–19; male mentors/staff (18–40), all from Alexandra, affiliated with GRS. |
| **Data Collection** | |
| 17. Interview guide: Were questions, prompts, guides provided by the authors? | Yes, semi-structured guides pretested and refined; included in Annex. |
| 18. Repeat interviews: Were repeat interviews carried out? If yes, how many? | No repeat interviews reported. |
| 19. Audio/visual recording: Did the research use audio or visual recording? | Audio-recorded. |
| 20. Field notes: Were field notes made during and/or after the interview or focus group? | Yes, reflexive and analytic notes kept; transcripts reviewed. |
| 21. Duration: What was the duration of the interviews or focus group? | 45–70 minutes. |
| 22. Data saturation: Was data saturation discussed? | Yes, discussed: stopped at conceptual depth after 12 adolescent interviews. |
| 23. Transcripts returned: Were transcripts returned to participants for comment and/or correction? | No. Transcripts were not returned to participants for correction. |
| **Analysis and Findings** | |
| 24. Number of data coders: How many data coders coded the data? | Two coders. |
| 25. Description of the coding tree: Did authors provide a description of the coding tree? | Coding scheme described: 92 codes, 13 categories, themes refined iteratively. |
| 26. Derivation of themes: Were themes identified in advance or derived from the data? | Themes derived inductively from data. |
| 27. Software: What software, if applicable, was used to manage the data? | Atlas.ti (v23.1.0). |
| 28. Participant checking: Did participants provide feedback on the findings? | Yes, preliminary themes presented to staff in workshop (member checking). |
| 29. Quotations presented: Were participant quotations presented to illustrate the themes/findings? | Yes, quotations used throughout Results. |
| 30. Data and findings consistent: Was there consistency between the data presented and the findings? | Yes, data and findings consistent. |
| 31. Clarity of major themes: Were major themes clearly presented in the findings? | Yes, major themes clearly presented in tables and narrative. |
| 32. Clarity of minor themes: Is there a description of diverse cases or discussion of minor themes? | Yes, minor/diverse cases described (e.g., differing views on masculinity). |
