# Supplementary material for: “Being a man is like being put in a box”: A qualitative study of adolescent boys’ and young men’s understanding and experiences of mental health in an urban community in South Africa
Source: PLOS Ment Health. 2026 Feb 6;3(2):e0000365. doi: 10.1371/journal.pmen.0000365 (PMC12880673; doi:10.1371/journal.pmen.0000365)
Supplement: S2 File — (DOCX) [file pmen.0000365.s002.docx]

**Cover Sheet: IN-DEPTH INTERVIEW WITH PARTICIPANTS**

***Instructions to the facilitator:*** Complete the following information before the INTERVIEW. Read the introduction to the interviewee. After collecting initials, detach the cover sheet. Start the recorder and state the interview's date, time, and location aloud.

| **PARTICIPANT Name:** | **Date:** | **Start Time:**  **End Time:** |
| --- | --- | --- |
| **PARTICIPANT Initials:** | **Venue:** | **Moderator:** |

## INTRODUCTION

I want to thank you for taking the time to talk to me today. My name is Chris Barkley, and today we talk about your understanding and experiences of mental health.

Our conversation should take 45-60 minutes. I will record the discussion because I don’t want to miss any of your comments. Please be sure to speak up so that the recorder picks up your comments.

Your responses will be kept confidential, and any information I share will not identify you. You don’t have to talk about anything you don’t want to, and you may end the discussion at any point, but your experiences and ideas are valuable, so please share what you feel comfortable sharing.

Do you have any questions about what I have just explained?

## Turn on the voice recorder. Record the participant’s name and the interview's date, time, and location and begin the discussion.

**In-depth Interview: Participants**

| **Topic** | **Main questions** | **Follow-up questions** |
| --- | --- | --- |
| 1. Introduction | - Can you please start by introducing yourself and sharing a bit about your background? | - Can you tell me about your family and friends? - Can you tell me about what you like to do for fun? |
| 2. Mental Health Perceptions | - Can you tell me what comes to mind with the term mental health? - How would you define mental health in your own words? - Are there any stigmas or stereotypes you believe exist around mental health, particularly related to adolescent boys? - Could you share any personal experiences or observations regarding this matter? | - Where do you usually seek information or learn about mental health? - Have you encountered any difficulties in understanding mental health? |
| 3. Factors influencing mental health | - Can you tell me about the types of support systems you have, such as family, friends, or teachers? - How do these relationships affect your mental well-being? - Can you tell me about how life in Alexandra impact your mental health and well-being? - How do social media and technology affect your mental well-being? Have you had any negative or positive experiences related to social media? - What are your experiences in school like? Do you feel the school environment promotes mental well-being? Are there any stressors or factors that may contribute to your mental health while at school? | - Do you admire any role models or individuals who positively impact your mental well-being? - What qualities or behaviours make them influential to you? |
| 4. Coping strategies | - How do you typically cope with stress or difficult emotions? - Are there any specific activities like sports, hobbies, or creative outlets that help you maintain good mental well-being? - Can you share your experiences with expressing your emotions? - Do you feel comfortable sharing your feelings with others? | - Do you believe society expects you to hide or suppress your emotions? |
| 5. Help seeking and resources | - What are your perceptions of seeking help for mental health concerns? - Have you ever sought support before? If so, did you face any barriers or challenges in doing so? | - Are you aware of any mental health resources available to you, such as counseling services, helplines, or community programs? - Have you ever utilized any of these resources? If yes, could you share your experiences? |
| 6. Masculinity | - What are some of the expectations and perceptions (masculine norms) people have about how men and boys? - Are there any specific ways in which you feel pressured to conform to ideas about how men and boys should be that affect you (your mental health and well-being)? - Have you ever felt the need to hide or suppress your emotions because of societal expectations related to masculinity? Can you share any experiences or examples? - Do you think there are certain emotions or struggles that are typically associated with being a man or being a woman? How does this influence how you perceive and express your own emotions? - Are there any barriers or challenges you face in seeking help or support for mental health concerns due to societal expectations around masculinity? - What do you think can be done at a societal level to create a more supportive and understanding environment for boys and young men in terms of mental health? | - Are there any role models or examples in popular culture or your community that challenge traditional notions of masculinity and promote a healthier approach to mental health? - How do these examples influence you? |
| 7. Change over time | - Can you tell me about any significant life transitions or events that impacted your mental well-being? - How has your well-being changed over time? | - Have you noticed any changes or shifts in societal attitudes towards mental health? If yes, in what ways? |
| Closing | - Is there anything else you would like to share related to the topic? | Thank you for your time! |

| **Probes** |
| --- |
| *Example Probes:*  You mentioned_______can you tell me a bit more about that?  That is interesting. How did you feel when______was happening?  Could you tell me about your thoughts about_________?  You mentioned________. What did you mean by that?  How did you act further on the_____________experience you mentioned?  Can you share any experiences or examples of _____________?  Can you tell me about an experience that illustrates your point about_____________? |
|  |
|  |
