# Supplementary material for: “Being a man is like being put in a box”: A qualitative study of adolescent boys’ and young men’s understanding and experiences of mental health in an urban community in South Africa
Source: PLOS Ment Health. 2026 Feb 6;3(2):e0000365. doi: 10.1371/journal.pmen.0000365 (PMC12880673; doi:10.1371/journal.pmen.0000365)
Supplement: S3 File — (DOCX) [file pmen.0000365.s003.docx]

**In-Depth Interview Guide**

Participants

**Topic: Introduction**

*About You*

- What’s your name, age, and where do you live?
- Who do you live with at home?
- Tell me a bit about your family and friends.
- What school do you go to?
- What do you like to do for fun?

*Life in Alexandra*

- What do you like about growing up in Alex?
- What’s difficult or frustrating about life here?
- What do boys your age usually do for fun?

*Being a Teenager*

- What do you enjoy about being a teenage boy?
- What’s challenging about it?
- Do you feel understood by the people around you?
- What excites you about your future?
- What worries you about it?

**Topic: Mental Health**

- Have you heard of the term “mental health”? What do you think it means?

*When I say mental health, I’m talking about things like your feelings and emotions, your thoughts, and your actions.*

- - What does it mean to you? How would you describe mental health?
  - What are your thoughts on mental health issues among teenagers?
- Where have you learned about mental health (e.g., school, home, media)?
- Can you think of a time something affected your mood or how you saw yourself?

**Topic: Coping and Support**

- Are there things in your life that make you feel stressed, sad, or angry?
- What do you do when you’re feeling down or stressed?
- Do you talk to anyone when things are hard? Who?
- Do you feel comfortable sharing your feelings?
- What would you say to a friend who wasn’t doing well?

**Topic: Social / Environment**

*School Life*

- What do you like and not like about school?
- Have you ever been bullied? How did you handle it?
- Do you think school helps or harms your well-being?

*Family and Home*

- How does your family make you feel about yourself?
- Who supports you most—at home or outside?
- Do you think your home is a supportive place?

*Social Media*

- Are you on social media? What do you use it for?
- How do your friends influence your choices or how you see things?

*Role Models*

- Who do you look up to—someone in your family, community, or a celebrity?
- What do you like about them?

**Topic: Gender Expectations**

- Do boys and girls face the same problems?
- Do they handle stress in the same way?
- What’s expected of you as a boy?
- What does “act like a man” mean to you?

**Topic: Getting Help**

- If you needed help, would you feel comfortable talking to someone about mental health?
- If you could ask for any kind of support for your mental health, what would it be?

**Final Thoughts**

- Is there anything else you want to share about this topic?

| **Probes** |
| --- |
| *Example Probes:*  You mentioned_______can you tell me a bit more about that?  That is interesting. How did you feel when______was happening?  Could you tell me about your thoughts about_________?  You mentioned________. What did you mean by that?  How did you act further on the_____________experience you mentioned?  Can you share any experiences or examples of _____________?  Can you tell me about an experience that illustrates your point about_____________? |
