# Supplementary material for: “Being a man is like being put in a box”: A qualitative study of adolescent boys’ and young men’s understanding and experiences of mental health in an urban community in South Africa
Source: PLOS Ment Health. 2026 Feb 6;3(2):e0000365. doi: 10.1371/journal.pmen.0000365 (PMC12880673; doi:10.1371/journal.pmen.0000365)
Supplement: Attachment — Submitted filename: Response to Reviewer.docx [file pmen.0000365.s004.docx]

Dear Reviewer,

We sincerely thank you for your thoughtful and constructive comments, which have significantly strengthened this manuscript. Your feedback helped us improve the clarity, depth, and coherence of the paper, particularly in the discussion, methodological justification, and actionable recommendations. We have carefully addressed each comment, as detailed in the responses below, and believe the revisions enhance both the scholarly and practical contributions of the study.

| **Reviewer’s Comment** | **Author Response** |
| --- | --- |
| The manuscript lacks a conclusive section and actionable recommendations. Adding these would strengthen the paper's impact (line no. 47). | We have strengthened the Conclusion to clearly summarize the study’s key findings and their implications and provided more actionable recommendations for intervention design targeting adolescent boys and young men in Alexandra. We worked to ensure that our recommendations are grounded in our results and aligned with current literature. |
| The use of purposive sampling is noted, but justification for its selection over convenience sampling or data saturation is required. Please clarify the rationale behind this choice (line no. 146). | We have expanded the Methods section to justify the purposive sampling, explaining that it was chosen to obtain rich, context-specific data from those with relevant lived experiences. We further explained our decision to stop interviews based on conceptual depth rather than saturation. |
| The results section should follow the order of objectives outlined in the introduction for better coherence and flow (line no. 209). | We have re-orded the three study aims presented in the Introduction, ensuring clearer coherence and flow between the aims, Results, and Discussion sections. Each aim is presented with its corresponding themes and supporting participant quotes. |
| The discussion section is underdeveloped. Each result should be thoroughly discussed in this section to provide depth and context (line no. 430). | We have substantially expanded the Discussion to link each main result to relevant literature, both South African and international. We explicitly interpret the meaning of each finding in context, highlighting how our study adds to the current evidence base. |
| The discussion lacks reflective insights. Incorporating reflections would add value to the manuscript (line no. 430). | We have incorporated reflections into the Discussion and added a Reflexivity section to the Methods. We described how the research team’s backgrounds, identities, and relationships with participants may have influenced data collection and interpretation, and how these perspectives shaped the analytic process. |
| The manuscript does not adequately address the limitations of the study. A detailed discussion on limitations is essential for transparency and credibility (line no. 502). | We added a Limitations section to include a detailed account of methodological and contextual limitations, including potential social desirability bias, translation challenges, and the limited generalisability of findings beyond the study setting. |
| The references cited are outdated. It is recommended that at least 90% of the references be from the period between 2015 and 2024/2025 to reflect current research trends and findings (line no 521). | We reviewed and updated the reference list to ensure at least 90% of sources are from 2015–2025, adding recent studies and reviews where applicable, while retaining a few key seminal works where necessary. |
