# Supplementary material for: “Being a man is like being put in a box”: A qualitative study of adolescent boys’ and young men’s understanding and experiences of mental health in an urban community in South Africa
Source: PLOS Ment Health. 2026 Feb 6;3(2):e0000365. doi: 10.1371/journal.pmen.0000365 (PMC12880673; doi:10.1371/journal.pmen.0000365)
Supplement: Attachment — Submitted filename: Response_to_Reviewer_auresp_2.docx [file pmen.0000365.s005.docx]

Dear Reviewer,

We sincerely thank you for your constructive feedback and for recommending the inclusion of a completed COREQ (Consolidated Criteria for Reporting Qualitative Research) checklist. In response to your comment, we have made the following revisions:

1. **COREQ checklist:** We have completed the 32-item COREQ checklist and provided it as Supporting Information (Appendix A)
2. **Minor revision to the Methods.** We added the following sentence to the Research design subsection to clarify our adherence to reporting standards: “To enhance transparency and completeness of reporting, the study methods and results were mapped to the Consolidated Criteria for Reporting Qualitative Research (COREQ) checklist [37], with the completed checklist provided as Supporting Information.”
3. **Clarifications for alignment with COREQ**
   a. We clarified the prior relationships between participants and the research team, noting that the adolescent boys did not know the researchers, while GRS youth mentors and staff were familiar with them through their organizational roles.
   b. We clarified how coders handled disagreements, noting that, while no major disagreements arose, the two researchers discussed their different perspectives and highlighted what felt most important in the data.

We believe these revisions fully address the reviewer’s request and strengthen the transparency of our reporting.

With thanks and kind regards,

Christopher Barkley (on behalf of all co-authors)
