# Supplementary material for: “Being a man is like being put in a box”: A qualitative study of adolescent boys’ and young men’s understanding and experiences of mental health in an urban community in South Africa
Source: PLOS Ment Health. 2026 Feb 6;3(2):e0000365. doi: 10.1371/journal.pmen.0000365 (PMC12880673; doi:10.1371/journal.pmen.0000365)
Supplement: Attachment — Submitted filename: Response to Reviewers.docx [file pmen.0000365.s006.docx]

Dear Reviewer/Editor,

We sincerely thank you for your thoughtful and constructive comments, which have significantly strengthened this manuscript. Your feedback helped us improve the clarity, depth, and coherence of the paper, particularly in the discussion, methodological justification, and actionable recommendations. We have carefully addressed each comment, as detailed in the responses below, and believe the revisions enhance both the scholarly and practical contributions of the study.

| **Reviewer Comment** | **Author Response** |
| --- | --- |
| The paper would benefit from incorporating additional interview excerpts from the adolescent participants to more clearly substantiate the analytical claims made in the analysis and discussion. Greater use of participants’ own words would strengthen the depth of the arguments. | We agree that greater use of participants’ own words strengthens the analysis. We incorporated additional direct quotations in the Results sections. |
| The authors discuss masculine norms as though they are fixed and uniform. However, constructions of masculinity are fluid, relational, and contextually produced. Social, cultural, and structural factors play a significant role in shaping how boys and men construct their masculine identities | We appreciate this important clarification. The Discussion section had touched the fluid nature of masculine norms [...*many rejected these as “old” or “traditional”, instead sharing views that encouraged “sharing your feelings”, “asking for help”, and “showing emotions because we’re all human.” As masculine norms change, so do their meaning and health implications for boys [13], and the participants’ voiced beliefs that possibly signal shifts in attitudes and a desire for redefining masculinity, something found in other studies]*language has been added to emphasize how social, cultural, historical, and structural factors shape masculinity. See the revised Introduction and Discussion sections. |
| Including an early section that outlines the nature of masculinity and identifies the forms of masculinity that are hegemonic within the Alexandra context would provide a stronger conceptual foundation for the arguments that follow. | We strengthened the conceptual framing in the Introduction by including an expanded discussion of masculinity, including hegemonic masculinity as it operates within South Africa and specifically within economically marginalized urban contexts such as Alexandra. See the Introduction. |
| In the analysis and discussion, the authors appear to suggest a unidirectional relationship in which masculinity shapes mental health, as though masculinity pre-exists mental health. However, mental health experiences often precede and actively inform the construction of masculine identities. It would therefore strengthen the paper for the authors to also consider how the boys’ mental health experiences shaped, mediated, and were implicated in the ways they understood and performed masculinity | We agree with this point and revised the Discussion section to explicitly address the bidirectional relationship of mental health and masculinity, providing illustrative examples. |
| Some of the boys asserted that mental health issues are for White people. This points to important racialised understandings of mental health that warrant deeper analysis. Given South Africa’s historical and socio-political context, a more sustained discussion of the racial overtones shaping how mental health is perceived, accessed, and legitimised would significantly strengthen the paper | We thank the reviewer for highlighting this critical issue. We revised the Introduction and Discussion and added a recommendation for intervention design to more fully explore how culture, socio-political, spiritual, and racial belief systems influence mental health and masculinity. We also strengthened engagement with the Alexandra context, and believe these dynamics are now more explicitly integrated into the manuscript. See Introduction, Discussion, and Recommendation 6. |
| While the authors briefly note this complexity in the conclusion, the paper does not sufficiently engage with the nuanced realities of the Alexandra context, particularly the interplay of varied racial and cultural belief systems in the construction of masculinities and the intersection with mental health, especially, in the analysis and discussion. | We thank the reviewer for highlighting this critical issue. We revised the Introduction and Discussion and added a recommendation for intervention design to more fully explore how culture, socio-political, spiritual, and racial belief systems influence mental health and masculinity. We also strengthened engagement with the Alexandra context, and believe these dynamics are now more explicitly integrated into the manuscript. See Introduction, Discussion, and Recommendation 6. |
| Providing a brief background of the adolescent participants would add depth and contextual richness to the subsequent analysis, allowing readers to better situate the boys’ experiences and narratives. | We appreciate this suggestion. The manuscript already includes detailed eligibility criteria and contextual information about participants. We did not add further background details beyond what is already included. |
| How many interviews were conducted with each of the applicants. | We have clarified that one individual, semi-structured interview was conducted with each participant. See Data Collection. |
| In addition to clinical ethical considerations, the authors should clarify what other safeguards were put in place to protect participants from potential emotional or psychological harm, considering the sensitive and personal topics explored in the study. | We agree that this is an important point. However, we believe the information provided about referral protocols, consent, and safeguarding/support mechanisms are sufficient. See Ethical Considerations. |
